# The Impact of Socioeconomic Factors on Long-Term Mortality Associated With Exposure to PM_2.5_: A Systematic Literature Review and Meta-Analysis

**DOI:** 10.3389/phrs.2025.1607290

**Published:** 2025-03-26

**Authors:** Henrik Olstrup, Wasif Raza, Johan Nilsson Sommar, Hans Orru

**Affiliations:** Department of Epidemiology and Global Health, Umeå University, Umeå, Sweden

**Keywords:** air pollution, socioeconomic status, mortality, education, income, lifestyle

## Abstract

**Objectives:**

Socioeconomic status (SES) is in many cases related to air pollution exposure, but less is known about its effects on susceptibility to air pollution. The main aim of this study was to analyse the impact of SES on health effects associated with exposure to fine particles (PM_2.5_).

**Methods:**

Firstly, a systematic literature review of studies analysing the impacts of SES on health effects related to air pollution exposure was carried out. Secondly, a meta-analysis was performed by analysing studies on long-term mortality associated with exposure to PM_2.5_ divided into different SES groups.

**Results:**

The meta-analysis showed that the relative risk (RR) for all-cause mortality associated with PM_2.5_ did not depend on individual education or income. It also revealed that adjustment for individual lifestyle factors (such as smoking, alcohol intake, physical activity, eating behaviours, and body mass index), in addition to adjustment for SES, did not significantly change the RR.

**Conclusion:**

The association between all-cause mortality and PM_2.5_ did not depend on education or individual income. Due to the high heterogeneity observed, further studies are required to draw firm conclusions.

## Introduction

Exposure to air pollution is known to have detrimental health effects. According to the World Health Organization, ambient air pollution is estimated to have caused around 4.2 million premature deaths worldwide in 2019 [1]. An increased risk of premature mortality associated with long-term exposure to PM_2.5_ has been shown in several meta-analyses e.g., [2–5]. Several studies have documented unequal environmental exposures by ethnicity and economic class [6–8], but there is limited evidence for an association between socioeconomic status and air pollution-related health effects.

**FIGURE 1:**
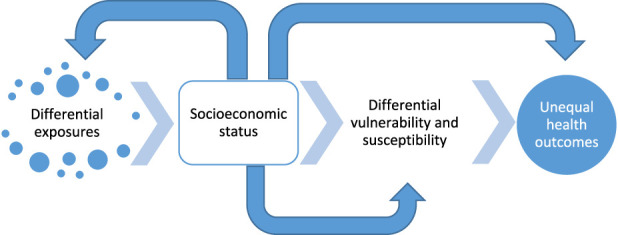
Potential pathways by which socioeconomic status can increase exposure, vulnerability, and susceptibility to air pollutants in relation to unequal health outcomes (adapted from [12]) (Umeå, Sweden. 2024).

Socioeconomic status (SES) is defined as a collective term for the quantification of social and economic status, and it tends to be associated with better health [9]. SES can significantly influence the state of health, and poor health is associated with lower education, less work capacity, and thereby lower earned income. There are three main factors contributing to the SES-health relationship: (1) the ability to access health-promoting resources and treatments; (2) differences in health habits and lifestyle factors; and (3) the reverse relationship, namely that less healthy individuals generally have less education, work less, and earn lower incomes [9, 10]. SES can potentially influence health outcomes related to air pollutants in several ways. In general, people of lower SES may experience greater effects related to exposure to air pollutants as they tend to live closer to traffic and industries, have a higher degree of occupational exposure, and have less access to healthcare [10]. Apart from poorer ambient air quality, households of low SES are usually linked to low-quality housing (poorer building quality, poorer ventilation, and less living space), second-hand indoor smoke, and higher occupant density resulting in greater resuspension of particles [11]. The potential pathways by which SES can increase both susceptibility and vulnerability to air pollutants are presented in Figure 1.

The relationship between SES and air pollution exposure depends on several factors. A general pattern with higher exposure to air pollutants among lower SES groups has been found in a number of studies conducted across North America, Europe, and South America [8, 13–27]. However, when the relationship between SES and air pollution exposure was analysed at both urban and rural locations in a mother-child cohort in France, higher exposure in lower SES groups was found in urban areas, while a U-shaped relationship was found in rural areas [22]. Differences in the relationships between SES and the concentrations of PM_2.5_, depending on urban or rural locations, were also observed in India, where several factors of SES were analysed [28].

In a systematic review by Hajat et al. [29], the variations in air pollutant concentrations among different SES groups were examined in more detail. Specifically, regarding SES and exposure to PM_2.5_ and NO_2_, a large number of studies conducted in North America found lower concentrations of these pollutants among higher SES areas/groups/individuals [30–44]. Mixed associations between SES and exposure to PM_2.5_ and NO_2_, both positive and negative, were found in four studies [45–48], and two studies reported no association [37, 42]. However, mixed results were reported on the relationship between exposure to ozone and SES, with four studies showing higher ozone concentrations among higher SES areas/groups/individuals [30, 35, 37, 40], and in two studies a positive association was obtained, which was similar for PM_2.5_ and NO_2_ [49, 50].

In comparison with Europe and North America, the relationship between SES and air pollution exposure is somewhat different in Asia. When the association between SES and air pollutants (PM_2.5_ and NO_2_) was analysed across China (both urban and rural residents), higher SES was associated with higher concentrations of air pollutants [51]. However, the relationships between SES and air pollution exposure varied within China, revealing contradictory findings in certain contexts [52, 53]. In Korea, individuals who belonged to small families were less educated, resided in areas with a higher neighbouring index (calculated using municipality-level data on occupation, population density, and number of service industries), and lived in municipalities with better air quality [54].

Despite an overall pattern of higher air pollution concentrations among lower SES groups in the Western world, the assumption of more serious health effects associated with air pollution exposure needs to be investigated. When years of life lost attributed to PM_2.5_ in England were analysed based on differences in SES, the pattern of PM_2.5_ concentrations made only a small contribution to the SES gradient [21]. Nevertheless, regarding air pollution and SES, a small number of studies focusing on both short- and long-term effects have reported more serious health effects among lower SES groups. For instance, an increased risk of all-cause and cardiovascular mortality among low SES groups was suggested in a time-series study based on a cohort in Canada [55]. In a cross-sectional study in the U.S., PM_2.5_-related mortality was higher in census tracts with the lowest SES [56]. Based on an ecological study conducted in Wales, air pollution concentrations were highest in deprived areas, and air pollution added to deprivation-health associations in terms of increased mortality [57]. Higher hazard ratios in lower SES neighbourhoods were also observed when the association between long-term exposure to PM_2.5_ and cardiovascular disease was analysed in participants from the Women’s Health Initiative Observational Study [58].

SES effects have also been associated with increased morbidity. An independent effect of air pollution on expiratory volume was found among men in lower SES groups in a health survey in England [59]. On the other hand, based on a cross-sectional study of three European multicentre adult cohorts, the association between lung function and exposure to NO_2_ was not substantially modified by SES variables [60]. Regarding children, indications of greater effects of air pollution on respiratory health among lower SES groups have been found [61], as well as greater effects on asthma exacerbations and asthma-related hospitalizations [62]. Regarding the population older than 65 years, specific causes of death were more prevalent among certain SES groups, but long-term PM_2.5_ exposure did not significantly affect these relationships [63].

In the present study, a literature review and a meta-analysis were performed. The literature review was based on a compilation of previous studies addressing the impact of SES on health effects related to air pollution. In the meta-analysis, the impact of SES on long-term mortality was analysed. By analysing studies on mortality associated with long-term exposure to PM_2.5_, separated into different determinants of SES, such as level of education and income, the main purpose of this work was to evaluate whether SES status might affect population susceptibility to PM_2.5_-related mortality. A secondary aim was to assess the remaining confounding effect of individual lifestyle factors (such as smoking, alcohol intake, physical activity, eating behaviours, and body mass index) after adjustment for SES.

## Methods

### Study Design

To identify relevant studies concerning the association between long-term air pollution exposure and all-cause mortality, with an emphasis on SES factors, we first focused on recently published systematic reviews. We selected the publication by Chen and Hoek [4] as the basis for our analysis. An independent search through PubMed was conducted to find additional studies since the publication of Chen and Hoek [4]. The literature search was conducted according to the complete Preferred Reporting Items for Systematic Reviews and Meta-Analysis (PRISMA) checklist [64].

### Search Strategy and Selection Criteria

In the PubMed search, we used the same search terms as those in the review by Chen and Hoek, which included studies up to October 2018. Our updated search extended the period from July 1, 2018, to May 16, 2023, to capture more recent publications (Supplementary Table SA1). We applied the following inclusion criteria:

1. Studies using prospective and retrospective cohort study designs, as well as case-control and nested case-control study designs.
2. Studies assessing the impact of air pollution on the general population.
3. A description of the method for assigning exposure is included.
4. Studies on associations between long-term exposure to outdoor PM_2.5_ and mortality.
5. Information on population characteristics is provided.
6. Studies reporting the effect estimates in terms of relative risk (RR), odds ratio (OR), or hazard ratio (HR) with a 95% confidence interval (CI).
7. Studies written in the English language.

Studies of long-term effects on mortality associated with exposure to PM_2.5_, including individual SES factors as effect modifiers, were selected to be used for our meta-analysis. Educational level and income level were used as markers of SES. Studies reporting RRs with adjustment for SES followed by adjustment for lifestyle factors were also selected for the meta-analysis to determine whether any confounding lifestyle factors remained after adjustment for SES. The analysis considering the lifestyle factors is of high interest for the interpretation of results. The lifestyle factors that were included in the studies were fruit and vegetable intake, physical activity, smoking, alcohol consumption, and body mass index (BMI). The SES factors that were included in these studies were income, educational level, employment, ethnicity, marital status, urbanization, SES-based deprivation index, airshed, visible minority identity, indigenous identity, and immigrant status. Studies using an indirect adjustment for lifestyle factors were also included in the meta-analysis. When multiple studies were available from the same cohort, we considered only the most recent article. Studies reporting the effects of indoor air pollution were excluded. Studies in which PM_2.5_ concentrations were calculated from other exposure metrics, such as total suspended particles (TSP), were not included. Studies focusing on air pollution effects on specific population subgroups, such as patients or children, were also excluded.

From each study that met the inclusion criteria, we recorded information regarding author name, publication year, location, study cohort, study year, sample size, study design, follow-up duration, confounder adjustments, and effect modification by SES factors, including income and educational level. We extracted estimates from the most adjusted and the authors’ favoured models in cases where multiple estimates were reported in the studies. Two researchers (W.R. and H. Olstrup) have individually and independently screened all individual studies for potential relevance for further analysis, and any disagreement was resolved by two other researchers (J.S. and H. Orru).

### Statistical Analysis

In the analyses of SES factors, income was categorized into quintiles. Education was classified into three levels: primary (primary education or less), secondary (secondary school or equivalent), and tertiary (college or university). The lowest category in each variable was used as a reference in the statistical analyses. When studies used fewer SES categories, the same RR was applied to all categories corresponding to that categorization. We combined the effect estimates using fixed-effect meta-analyses to account for variability between estimates when two RRs corresponding to the specific categorization were presented in one original study. When the original study reported RRs with a different reference category, the RR was re-calculated, assuming that the RRs between categories were independent. The pooled RR for all-cause mortality and long-term exposure to PM_2.5_ was calculated using a random-effect meta-analysis employing the *metafor* add-on package in the R software. Effect estimates from individual studies were standardized for an increment of 10 μg m^−3^ PM_2.5_. Finally, publication bias was analysed using funnel plots (Supplementary Figure SA1).

## Results

### Selected Studies

**FIGURE 2:**
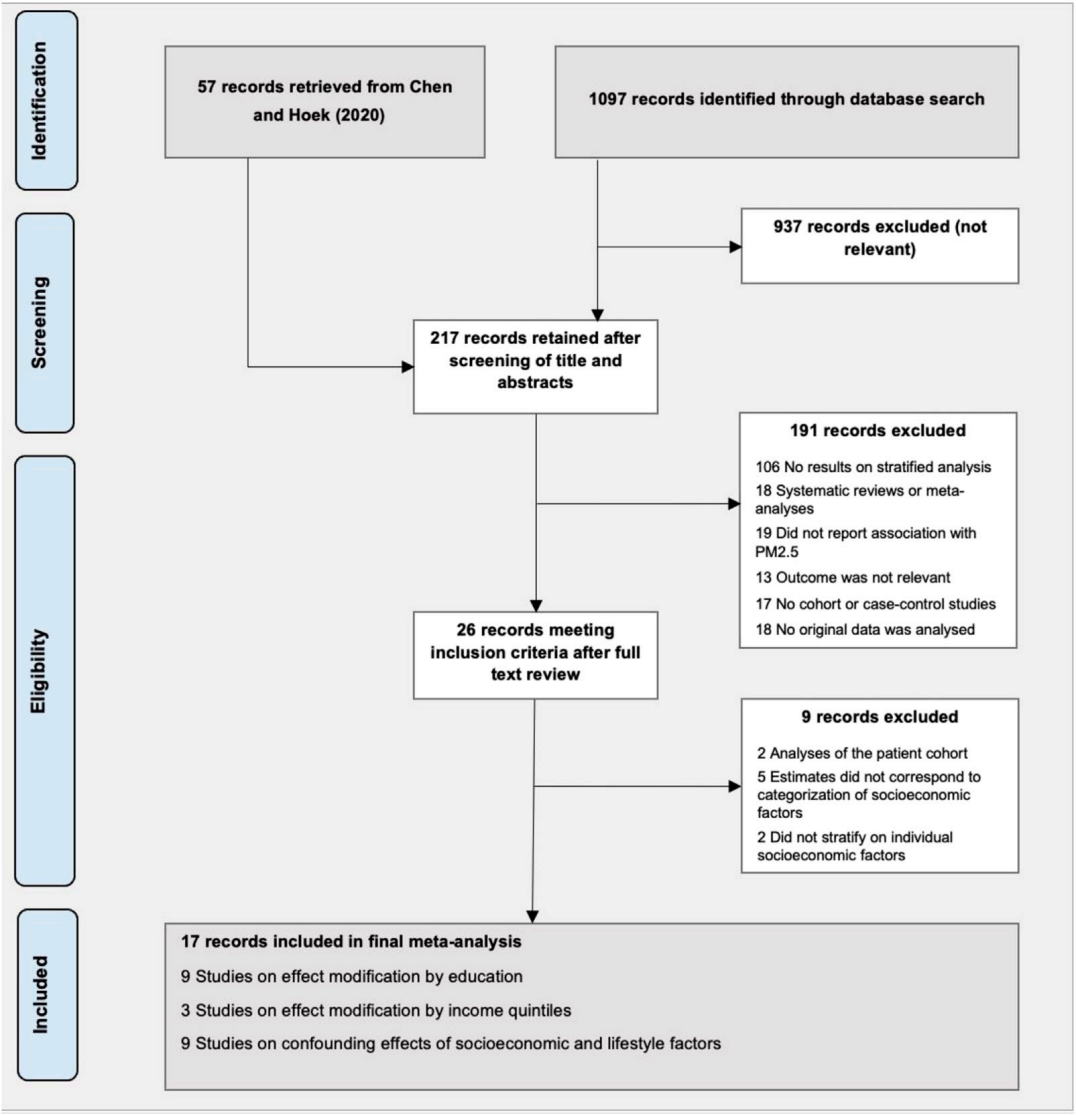
Flow chart of studies for inclusion in the meta-analysis (Umeå, Sweden. 2024).

The initial literature search yielded 1,097 articles, out of which 160 remained after screening the titles and abstracts. We further retrieved 57 records from the meta-analysis by Chen and Hoek [4], resulting in 217 records that were identified for full-text review. After screening the full texts, we further excluded 200 records and selected a total of 17 studies [65–81] for a meta-analysis of SES factors (Figure 2). Eight studies were conducted in North America, six in Europe, and three in Asia. Nine studies provided risk estimates stratified by individual-level education, and three studies by individual-level income. The remaining confounding effect due to individual-level lifestyle factors, after adjustment for individual-level SES factors, was assessed in nine studies. A detailed description of the characteristics of the studies included in the meta-analyses is presented in Supplementary Table SA2.

### Calculated Meta-Coefficients

**FIGURE 3:**
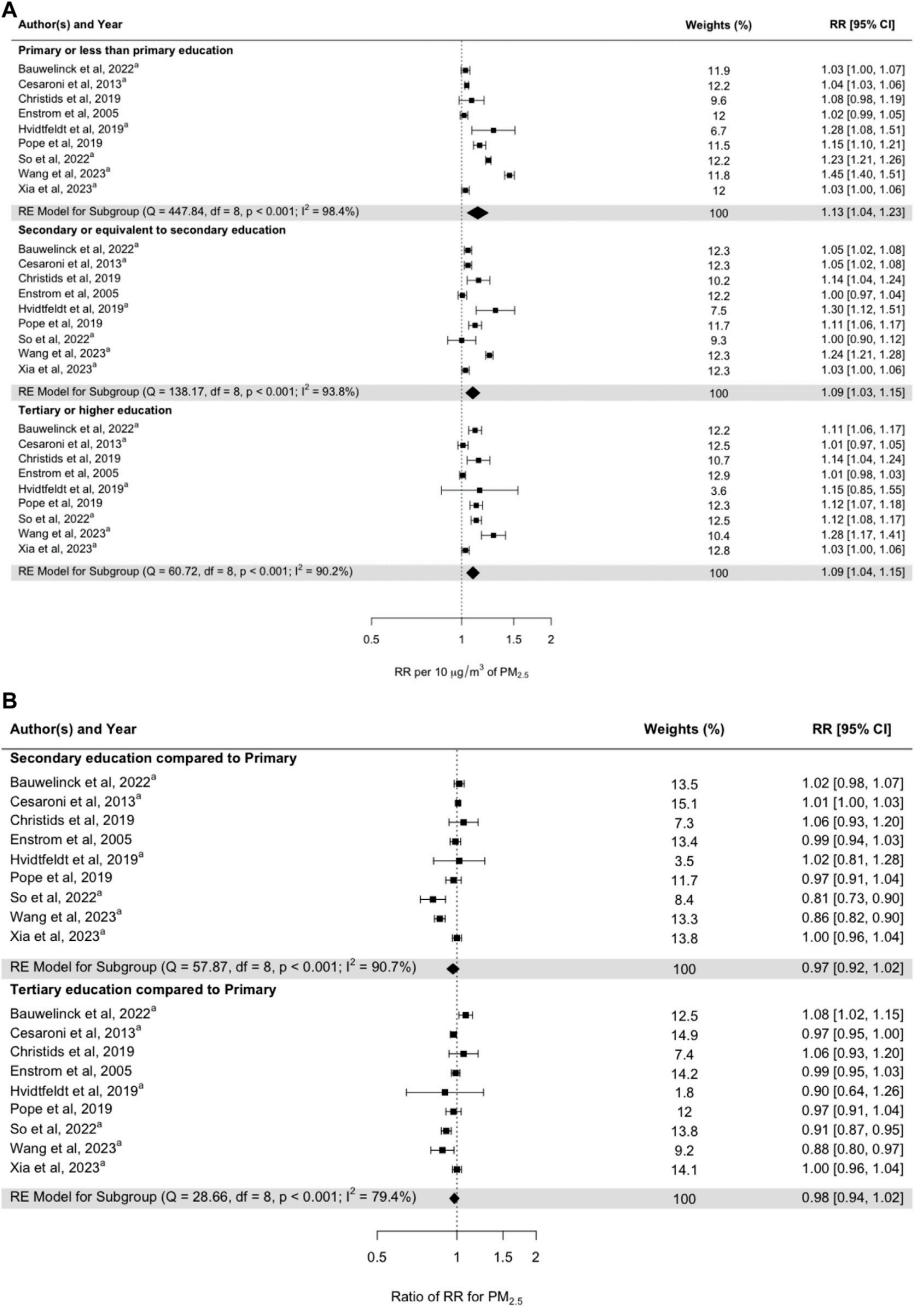
**(A)** Pooled random-effect meta-estimates are presented below the individual studies. **(B)** Subgroup risk estimates based on pooled mean differences in effect sizes between studies. Subgroups represent primary school education (at the top), secondary school education (in the middle), and tertiary school education (at the bottom). ^a^Studies examining the interaction effects of PM_2.5_ and education on all-cause mortality (Umeå, Sweden. 2024).

Although the point estimates of the meta-coefficients per 10 μg m^−3^ increase in PM_2.5_ were 1.13 (95% CI: 1.04–1.23), 1.09 (95% CI: 1.03–1.15), and 1.09 (95% CI: 1.04–1.15) in the subgroups of primary, secondary, and tertiary education, respectively (Figure 3A), the meta-analysis results did not indicate any difference in the size of the ratio of RR for all-cause mortality in relation to PM_2.5_ by individual-level education (Figure 3B).

**FIGURE 4:**
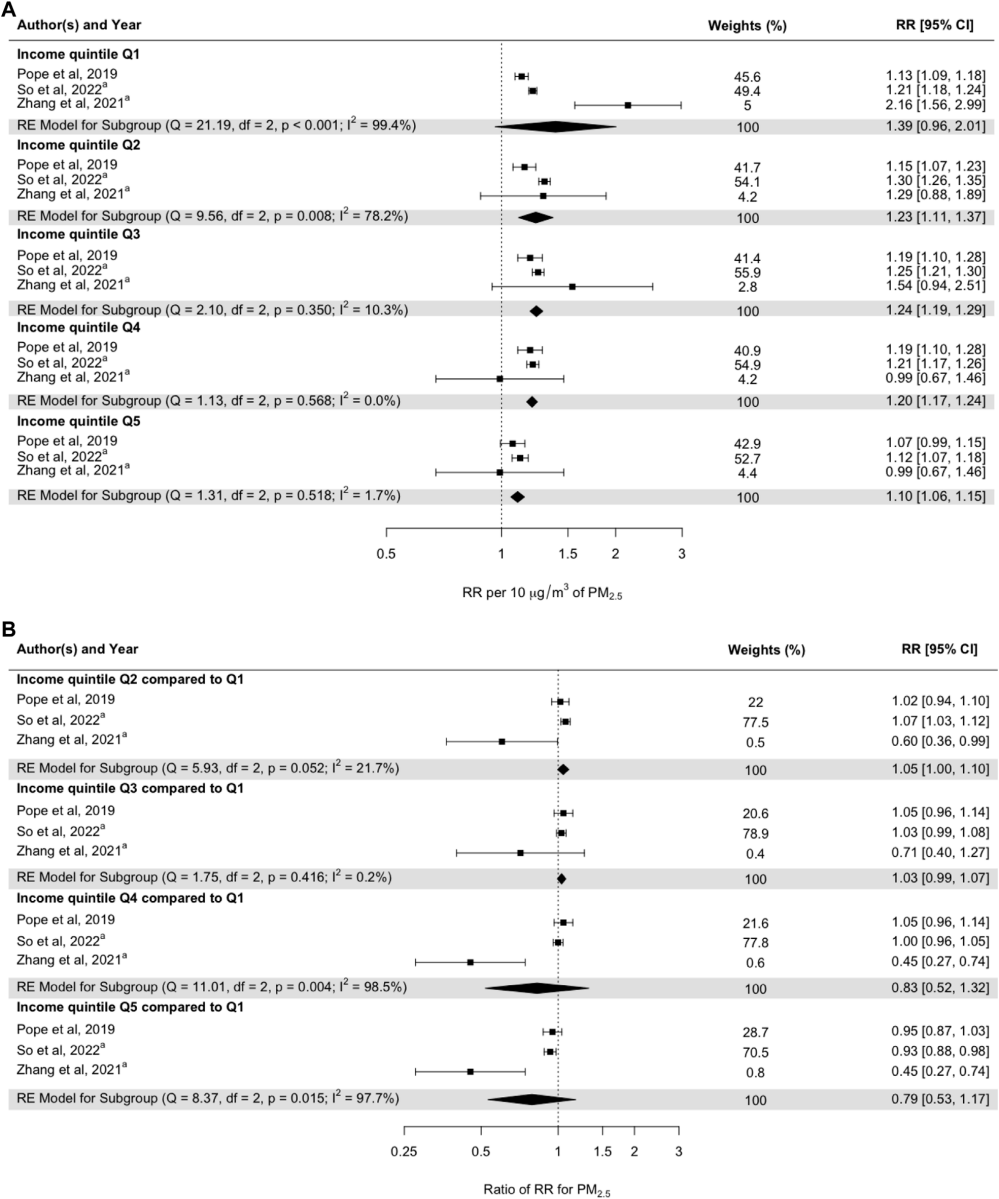
(A) Pooled random-effect meta-estimates are presented below the individual studies. (B) Subgroup risk estimates based on pooled mean differences in effect sizes between studies. Subgroups represent income quintiles, with Q1 representing the lowest income quintile and Q5 representing the highest income quintile. ^a^Studies examining the interaction effects of PM_2.5_ and individual-level income on all-cause mortality (Umeå, Sweden. 2024).

When the effect estimates were stratified by income quintiles, there was a gradient of a lower effect with increasing income quintile: 1.39 (95% CI: 0.96–2.01) 1st quintile, 1.23 (95% CI: 1.11–1.37) 2nd quintile, 1.24 (95% CI: 1.19–1.29) 3rd quintile, 1.20 (95% CI: 1.17–1.24) 4th quintile, and 1.10 (95% CI: 1.06–1.15) 5th quintile (effects per 10 μg m^−3^ increase in PM_2.5_) (Figure 4A). However, these risk estimates did not differ significantly when comparing quintiles 2, 3, 4, and 5 with quintile 1 (Figure 4B). The heterogeneity of the RRs when comparing the second and third quintiles with the first quintile was low, but it was high for the other quintiles.

**FIGURE 5:**
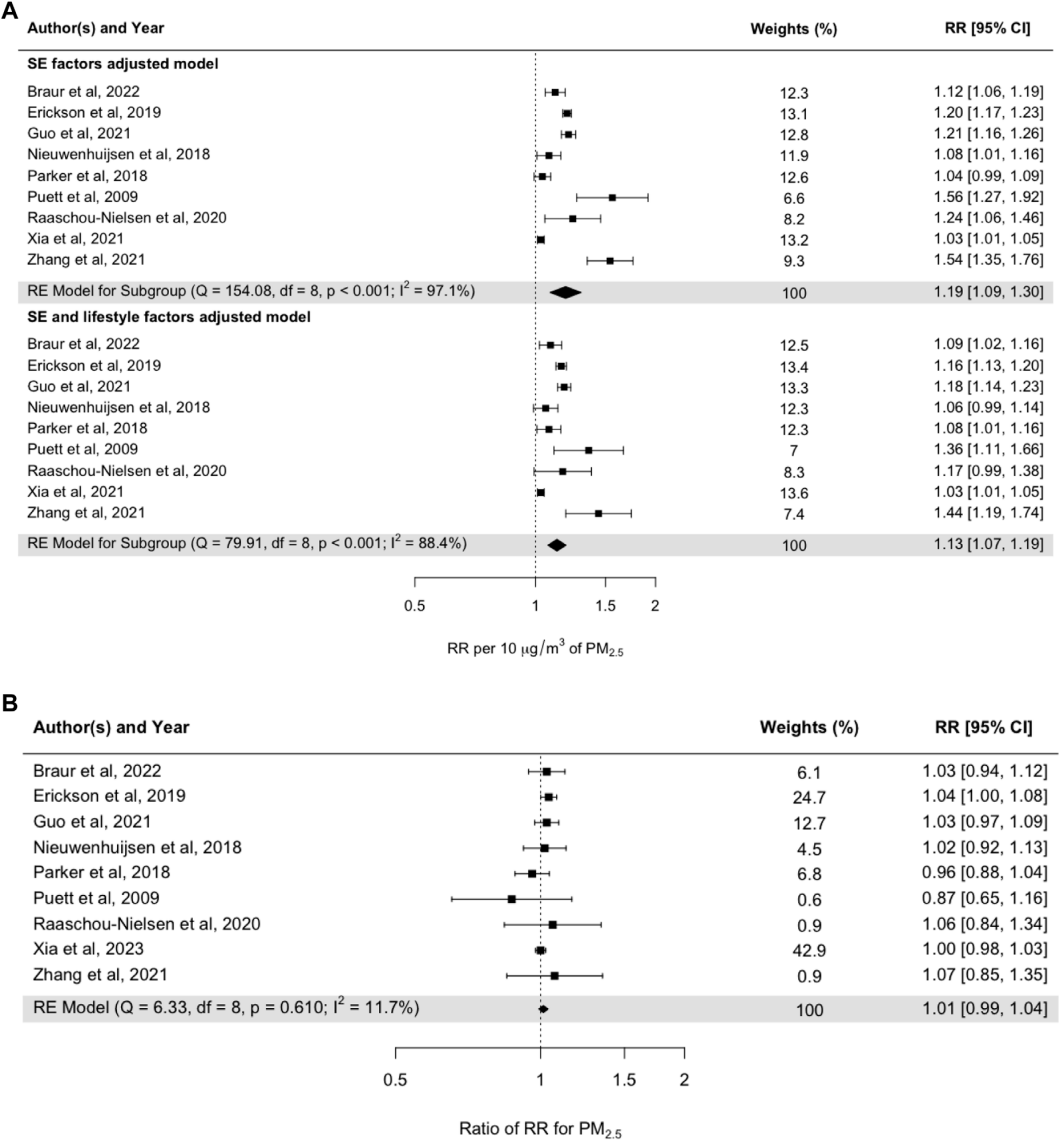
**(A)** Pooled random-effect meta-estimates are presented below the individual studies. **(B)** Subgroup risk estimates based on pooled mean differences in effect sizes between studies. Subgroups represent studies adjusted for SES factors and adjusted for both SES factors and lifestyle factors (Umeå, Sweden. 2024).

The confounding effect due to individual lifestyle factors was assessed in meta-analyses among studies reporting RRs with adjustment for individual-level SES factors, followed by adjustment for individual-level lifestyle factors (Figure 5). The meta-coefficient after adjustment for SES factors was 1.19 (95% CI: 1.09–1.30) per 10 μg m^−3^ increase in PM_2.5_, and after additional adjustment for lifestyle factors, it was 1.13 (95% CI: 1.07–1.19) (Figure 5A). However, the RRs adjusted for both individual lifestyle factors and SES were not statistically significant when compared to the RRs adjusted for SES factors only (Figure 5B). Heterogeneity between the studies was low (11.7%) when comparing RRs with and without adjustment (Figure 5B).

## Discussion

### Main Findings

The meta-analysis showed that the RRs for all-cause mortality associated with PM_2.5_ did not depend on individual education or income. The meta-analysis results also showed that adjustment for individual lifestyle factors in addition to adjustment for SES did not change the RRs. Out of the nine studies that were included in the meta-analysis by educational level, four were performed in Europe, three in North America, and two in China. One of the Chinese studies [78] and one Danish study [79] showed statistically significantly lower RRs for the groups with a relatively higher level of education, and a Belgian study [80] showed one statistically significantly higher RR for the group with a relatively higher level of education (Figure 3B). The other studies showed no statistically significant differences in mortality. Regarding the studies addressing the impact of SES in terms of differences in income (Figure 4B), one statistically significantly higher RR was found by So et al. [79] when comparing income group Q2 with income group Q1. However, the Canadian study [81] showed statistically significantly lower RRs in association with a higher income level when comparing Q5, Q4, and Q2 with Q1. The low number of studies presenting results by income certainly shows that more studies are needed. The fairly high number of subcategories that were used in the original studies reduces precision in the single estimate, but also increases the ability to assess a possible trend in the modifying effect of income.

A general pattern with higher air pollution concentrations among lower SES groups has been shown in Europe and North America [8, 13–22, 24–27], with somewhat different results in other areas [51–53, 82].

With generally higher air pollution concentrations among lower SES groups, it is reasonable to assume that a specific increase in PM_2.5_ (10 μg m^−3^) does not have as great effect on susceptibility to long-term mortality compared to lower baseline concentrations of air pollutants, especially in countries where air pollution concentrations are relatively low. This could explain the non-significant meta-coefficients when comparing higher versus lower educational levels based on studies conducted in the Western world (Figure 3B; Supplementary Table SA2).

Regarding SES and lifestyle factors, the lifestyle factors presented in Figure 5 were shown to have a low impact on the risk estimates for long-term mortality associated with exposure to PM_2.5_. The small impact of lifestyle factors on the risk estimates when included together with SES factors is somewhat unexpected and is very likely related to the small number of studies in the meta-analysis. The studies that were included were mainly carried out in developed countries.

### Results of This Study in Relation to Other Studies

The overall result of this study, namely an absence of clear differences in long-term mortality when comparing similar increases in PM_2.5_ across different SES groups, is not in line with several studies in this research area. Regarding air pollution, SES, and specific health outcomes, only a few studies have explored the potential mediating effect of SES factors on the association between ambient air pollution and specific health outcomes. In a time-series study based on a cohort in Canada, the associations between air pollution and mortality were analysed, and the participants were stratified into income quintiles. There were some indications of increased risks of all-cause and cardiovascular mortality at lower levels of SES [55]. In a cross-sectional study conducted in the U.S., the PM_2.5_ concentrations and the magnitude of the associations between PM_2.5_ and age-specific mortality were higher in the census tracts with the lowest SES [56].

Regarding lifestyle factors and their potential impact on the associations between air pollution exposure and SES, the results in this study indicated that lifestyle factors had only a small effect on the RR, without a statistically significant difference between including and not including lifestyle factors. Nevertheless, a previous study has found that occupational exposure, smoking, and outdoor air pollution are significant confounding factors for the association between SES (level of education) and respiratory health among women from the Ruhr area [83].

### Do Associations Depend on Vulnerability or Susceptibility?

The terms vulnerability and susceptibility refer to different aspects of how individuals or populations are affected. Vulnerability refers to a broader concept that encompasses the overall risk someone faces, and it is related to an increase in exposure. The reasons may be social, economic, and environmental: e.g., people living in impoverished areas may be more exposed and thus more vulnerable to the adverse effects of air pollution. Susceptibility is more specifically related to the degree to which a person’s health is affected by pollutants based on their physiological characteristics. For example, exposure to air pollution in young children as they are more sensitive to air pollution than adults because they breathe in more air per unit of body weight and, consequently, more pollution.

In Western countries, a general pattern with higher air pollution concentrations among lower SES groups has proven to be valid, while in China no relationship, or in some cases even an opposite relationship, has been found. Based on empirical evidence in the U.S., low-income and minority subpopulations are generally disproportionately exposed to higher pollution levels. With respect to physical (resource deprivation) and psychological stressors, an increased susceptibility to air pollution among lower SES groups has been suggested [84]. The impact of perceived air quality on self-rated health has also been shown to be more noticeable among lower socioeconomic groups [85], as well as among minorities [86].

Based on a review of European studies analysing the relationships between air pollution exposures among different SES groups, the general pattern was that subjects of low SES experienced greater health effects related to air pollution exposure regardless of the concentrations [87]. However, based on three European multicentre cohorts that were used to study the associations between NO_2_ concentrations and SES factors, a pooled analysis showed that participants with lower individual SES were exposed to lower levels of NO_2_, while participants living in neighbourhoods with a higher unemployment rate were exposed to higher concentrations. These patterns clearly demonstrate that individual and neighbourhood SES indicators capture different aspects of the association between SES and exposure to air pollution [88].

Heterogeneity in the relationships between NO_2_ concentrations and SES has been shown with respect to different cities in Sweden [89] and France [90]. Moreover, compared to area-level characteristics, individually measured SES characteristics were found to have larger effects related to air pollution among individuals belonging to lower SES groups [91].

Associations could be affected by indoor air pollution as well as ambient air pollution. Concerning indoor air pollution exposure among different SES categories, building characteristics, such as building quality, volume, ventilation system, occupant density, and occupant behaviour, may vary across different SES groups. A literature review based on 38 studies reported that low-SES individuals were exposed to higher indoor air concentrations of PM, NO_2_, volatile organic compounds, and second-hand smoke [11]. Worldwide, urban populations tend to spend approximately 90% of their time in different types of indoor environments [92], so exposure to indoor contaminants is an important predictor of population susceptibility to air pollution.

### The Possible Ways of Defining SES and the Choice of Income and Educational Level

The concept of SES is a broad term difficult to define. The most common markers of SES include income, poverty, wealth, education, occupation, income inequality, and subjective social status [93]. Income and educational level, which have been used in this meta-analysis, might not cover all aspects of the concept. Even though family income, educational attainment, and occupational status are considered as the “big three” operational definitions of SES [94], there are a number of limitations involved.

Hajat et al. [93] have pointed out that income captures the financial situation of households, but it is subject to both short- and long-term fluctuations, and there are further uncertainties associated with using household income as a marker of SES. Members of a household may have unequal access to the income [95], and, especially for retired individuals, household income may not reflect cumulative lifetime resources [96]. Moreover, as income level can modify health status earlier in life, the relationship between income and health may be subject to reverse causality [95].

Education commonly represents the number of years of education or the highest degree obtained. Both physical functioning and perceived health increase as a function of the number of years of education [97]. Education is usually carried out early in adulthood, and, in general, no reverse causation problems occur from linking education with health outcomes [95]. However, there are limitations and uncertainties associated with using education as a marker of SES: the quality of education varies regionally, the value of education has changed over time, and the level of education does not reflect career experiences [93, 95].

The high degree of heterogeneity, observed in this meta-analysis, could be at least partly explained by the uncertainties associated with using income and educational level as markers of SES, as mentioned above.

### Results of This Study and Their Implications for Policymaking and Future Research Needs

An important question in the context of air pollution exposure, SES, and their impact on health is to what extent differential exposure and differential susceptibility to air pollution among lower SES groups are relevant for addressing public health aspects and conducting health impact assessments.

The results presented in Figures 3, 4 show that the meta-coefficients for mortality associated with long-term exposure to PM_2.5_ were not significantly affected by SES factors related to individual educational and income levels. The long-term RRs adjusted for SES factors were also not significantly confounded by lifestyle factors (Figure 5).

In health impact assessment and health burden calculations, the choice of pollutant, SES factor, and geographical location may affect the results. Most of the studies included in the meta-analyses were performed in Europe and North America. The pattern with higher air pollution concentrations among lower SES groups has been shown to generally apply in the Western world. When comparing the effects due to a similar increase in PM_2.5_ among different SES groups, the relative effect associated with a 10 μg m^−3^ increase may not be so great in groups that are exposed to a relatively higher concentration. This could mean that comparisons based on the same increase in concentration do not capture possible differences in health effects linked to air pollution exposure among different SES groups. However, applying an average RR among all groups is certainly valid for calculating the impacts across a whole population.

How the issue of air pollution in different socioeconomic groups should be handled has been addressed in a few studies. When Chinese leaders declared war against air pollution in 2014, which means seriously addressing the problem of air pollution and actively working to take effective measures to reduce emissions, local authorities were tasked with addressing and incorporating socioeconomic factors [98]. Concerning citizens’ preferences for environmental protection in China, the populations from the lowest occupational class were least likely to mention environmental protection as a service into which more resources should be invested. Flatø [98] argued that from a policy point of view there is a need to consider social protection and the inclusion of environmental justice to enhance the interest in local environmental policy among lower SES groups in order to facilitate effective environmental governance. SES factors are also important in terms of opportunities and resources to reduce air pollution emissions and create better air quality. Different future scenarios in terms of improved air quality can be envisioned by considering SES factors, and they depend on differences in technological, institutional, and economic opportunities and limitations [99].

### Strengths and Limitations

A great challenge when analysing the health effects of air pollution exposure and the impact of SES is that air pollution is physical and measurable, whereas SES is relational and hard to quantify in a consistent way [100]. The main strength of this study is that studies investigating the relationship between mortality and long-term exposure to PM_2.5_, with SES as an effect modifier, have been selected through a careful and rigorous procedure following the PRIMSA guidelines for systematic reviews. The selected studies were carried out in different parts of the world, which is an advantage as different conditions prevail depending on geographical location. The main limitation of this analysis is the small number of studies that could be included in the meta-analysis. A high degree of heterogeneity was observed in our meta-analysis, which may restrict the generalizability of our findings. Moreover, PM_2.5_ was the only air pollutant included, and only educational level and income were used as markers of SES. More research on the effects of SES based on different air pollutants, total air pollution concentrations, SES factors, and geographical areas is needed to gain a better understanding of variations in susceptibility.

### Conclusion

The RRs for all-cause mortality associated with PM_2.5_ did not depend on education or individual income. Due to the high heterogeneity observed, further studies are, however, required to draw firm conclusions. Since adjustment for individual lifestyle factors did not change the RRs after adjustment for SES, cohort studies on only administrative registry data would need the same type of adjustment as traditional cohort studies based on examined (or surveyed) individuals. More research on the effects of SES based on different air pollutants, total air pollution concentrations, SES factors, and geographical areas is needed to gain a better understanding of how these factors are connected.
