# Supplementary material for: The Impact of Socioeconomic Factors on Long-Term Mortality Associated With Exposure to PM2.5: A Systematic Literature Review and Meta-Analysis
Source: Public Health Rev. 2025 Mar 26;46:1607290. doi: 10.3389/phrs.2025.1607290 (PMC11979636; doi:10.3389/phrs.2025.1607290)

**Appendix**

**Table SA1.** Search strategy in PubMed (Umeå, Sweden. 2024).

| **Search timeline:** 2018.07.01−2023.05.16 | | **Abstracts** |
| --- | --- | --- |
| #22 | (((((((((((particulate matter[Title/Abstract] OR particulate air pollution[Title/Abstract] OR PM[Title/Abstract] OR PM10[Title/Abstract] OR PM2.5[Title/Abstract]OR particles[Title/Abstract]) OR (Particulate Matter[nm])) OR (Particulate Matter[Mesh])) AND ((((Mortality[Mesh]) OR (mortality [Subheading])) OR (Death[Mesh])) OR (mortality[Title/Abstract] OR death[Title/Abstract]))) AND ((((Cohort Studies[Mesh]) OR (Case-Control Studies[Mesh])) OR (cohort[Title/Abstract] OR follow up[Title/Abstract] OR Longitudinal[Title/Abstract] OR Prospective[Title/Abstract] OR Retrospective[Title/Abstract])) OR (case-control[Title/Abstract]))) NOT ("Clinical Trial"[pt])) NOT ("animals"[Mesh:NoExp])) NOT ("Treatment Outcome"[MeSH])) NOT ("Air Pollution, Indoor"[Mesh])) NOT ("Occupational Exposure"[Mesh])) NOT ("time series"[Title])) AND (("2018/07/01"[Date - Publication] : "2023/05/16"[Date - Publication])) | 1,097 |
| #21 | ((((((((((particulate matter[Title/Abstract] OR particulate air pollution[Title/Abstract] OR PM[Title/Abstract] OR PM10[Title/Abstract] OR PM2.5[Title/Abstract]OR particles[Title/Abstract]) OR (Particulate Matter[nm])) OR (Particulate Matter[Mesh])) AND ((((Mortality[Mesh]) OR (mortality [Subheading])) OR (Death[Mesh])) OR (mortality[Title/Abstract] OR death[Title/Abstract]))) AND ((((Cohort Studies[Mesh]) OR (Case-Control Studies[Mesh])) OR (cohort[Title/Abstract] OR follow up[Title/Abstract] OR Longitudinal[Title/Abstract] OR Prospective[Title/Abstract] OR Retrospective[Title/Abstract])) OR (case-control[Title/Abstract]))) NOT ("Clinical Trial"[pt])) NOT ("animals"[Mesh:NoExp])) NOT ("Treatment Outcome"[MeSH])) NOT ("Air Pollution, Indoor"[Mesh])) NOT ("Occupational Exposure"[Mesh])) NOT ("time series"[Title]) | 2,708 |
| #20 | (((((((((particulate matter[Title/Abstract] OR particulate air pollution[Title/Abstract] OR PM[Title/Abstract] OR PM10[Title/Abstract] OR PM2.5[Title/Abstract]OR particles[Title/Abstract]) OR (Particulate Matter[nm])) OR (Particulate Matter[Mesh])) AND ((((Mortality[Mesh]) OR (mortality [Subheading])) OR (Death[Mesh])) OR (mortality[Title/Abstract] OR death[Title/Abstract]))) AND ((((Cohort Studies[Mesh]) OR (Case-Control Studies[Mesh])) OR (cohort[Title/Abstract] OR follow up[Title/Abstract] OR Longitudinal[Title/Abstract] OR Prospective[Title/Abstract] OR Retrospective[Title/Abstract])) OR (case-control[Title/Abstract]))) NOT ("Clinical Trial"[pt])) NOT ("animals"[Mesh:NoExp])) NOT ("Treatment Outcome"[MeSH])) NOT ("Air Pollution, Indoor"[Mesh])) NOT ("Occupational Exposure"[Mesh]) | 2,727 |
| #19 | ((((((((particulate matter[Title/Abstract] OR particulate air pollution[Title/Abstract] OR PM[Title/Abstract] OR PM10[Title/Abstract] OR PM2.5[Title/Abstract]OR particles[Title/Abstract]) OR (Particulate Matter[nm])) OR (Particulate Matter[Mesh])) AND ((((Mortality[Mesh]) OR (mortality [Subheading])) OR (Death[Mesh])) OR (mortality[Title/Abstract] OR death[Title/Abstract]))) AND ((((Cohort Studies[Mesh]) OR (Case-Control Studies[Mesh])) OR (cohort[Title/Abstract] OR follow up[Title/Abstract] OR Longitudinal[Title/Abstract] OR Prospective[Title/Abstract] OR Retrospective[Title/Abstract])) OR (case-control[Title/Abstract]))) NOT ("Clinical Trial"[pt])) NOT ("animals"[Mesh:NoExp])) NOT ("Treatment Outcome"[MeSH])) NOT ("Air Pollution, Indoor"[Mesh]) | 2,954 |
| #18 | (((((((particulate matter[Title/Abstract] OR particulate air pollution[Title/Abstract] OR PM[Title/Abstract] OR PM10[Title/Abstract] OR PM2.5[Title/Abstract]OR particles[Title/Abstract]) OR (Particulate Matter[nm])) OR (Particulate Matter[Mesh])) AND ((((Mortality[Mesh]) OR (mortality [Subheading])) OR (Death[Mesh])) OR (mortality[Title/Abstract] OR death[Title/Abstract]))) AND ((((Cohort Studies[Mesh]) OR (Case-Control Studies[Mesh])) OR (cohort[Title/Abstract] OR follow up[Title/Abstract] OR Longitudinal[Title/Abstract] OR Prospective[Title/Abstract] OR Retrospective[Title/Abstract])) OR (case-control[Title/Abstract]))) NOT ("Clinical Trial"[pt])) NOT ("animals"[Mesh:NoExp])) NOT ("Treatment Outcome"[MeSH]) | 3,015 |
| #17 | ((((((particulate matter[Title/Abstract] OR particulate air pollution[Title/Abstract] OR PM[Title/Abstract] OR PM10[Title/Abstract] OR PM2.5[Title/Abstract]OR particles[Title/Abstract]) OR (Particulate Matter[nm])) OR (Particulate Matter[Mesh])) AND ((((Mortality[Mesh]) OR (mortality [Subheading])) OR (Death[Mesh])) OR (mortality[Title/Abstract] OR death[Title/Abstract]))) AND ((((Cohort Studies[Mesh]) OR (Case-Control Studies[Mesh])) OR (cohort[Title/Abstract] OR follow up[Title/Abstract] OR Longitudinal[Title/Abstract] OR Prospective[Title/Abstract] OR Retrospective[Title/Abstract])) OR (case-control[Title/Abstract]))) NOT ("Clinical Trial"[pt])) NOT ("animals"[Mesh:NoExp]) | 3,400 |
| #16 | (((((particulate matter[Title/Abstract] OR particulate air pollution[Title/Abstract] OR PM[Title/Abstract] OR PM10[Title/Abstract] OR PM2.5[Title/Abstract]OR particles[Title/Abstract]) OR (Particulate Matter[nm])) OR (Particulate Matter[Mesh])) AND ((((Mortality[Mesh]) OR (mortality [Subheading])) OR (Death[Mesh])) OR (mortality[Title/Abstract] OR death[Title/Abstract]))) AND ((((Cohort Studies[Mesh]) OR (Case-Control Studies[Mesh])) OR (cohort[Title/Abstract] OR follow up[Title/Abstract] OR Longitudinal[Title/Abstract] OR Prospective[Title/Abstract] OR Retrospective[Title/Abstract])) OR (case-control[Title/Abstract]))) NOT ("Clinical Trial"[pt]) | 3,515 |
| #15 | ((((particulate matter[Title/Abstract] OR particulate air pollution[Title/Abstract] OR PM[Title/Abstract] OR PM10[Title/Abstract] OR PM2.5[Title/Abstract]OR particles[Title/Abstract]) OR (Particulate Matter[nm])) OR (Particulate Matter[Mesh])) AND ((((Mortality[Mesh]) OR (mortality [Subheading])) OR (Death[Mesh])) OR (mortality[Title/Abstract] OR death[Title/Abstract]))) AND ((((Cohort Studies[Mesh]) OR (Case-Control Studies[Mesh])) OR (cohort[Title/Abstract] OR follow up[Title/Abstract] OR Longitudinal[Title/Abstract] OR Prospective[Title/Abstract] OR Retrospective[Title/Abstract])) OR (case-control[Title/Abstract])) | 3,693 |
| #14 | (((Cohort Studies [Mesh]) OR (Case-Control Studies [Mesh])) OR (cohort[Title/Abstract] OR follow up[Title/Abstract] OR Longitudinal[Title/Abstract] OR Prospective[Title/Abstract] OR Retrospective[Title/Abstract])) OR (case-control[Title/Abstract]) | 4,035,942 |
| #13 | case-control [Title/Abstract] | 153,982 |
| #12 | cohort [Title/Abstract] OR follow up[Title/Abstract] OR Longitudinal[Title/Abstract] OR Prospective[Title/Abstract] OR Retrospective[Title/Abstract] | 2,872,650 |
| #11 | Case-Control Studies [Mesh] | 1,413,238 |
| #10 | Cohort Studies[Mesh] | 2,477,781 |
| #9 | (((Mortality [Mesh]) OR (mortality [Subheading])) OR (Death[Mesh])) OR (mortality[Title/Abstract] OR death[Title/Abstract]) | 2,219,327 |
| #8 | mortality[Title/Abstract] OR death[Title/Abstract] | 1,675,540 |
| #7 | Death[Mesh] | 163,678 |
| #6 | mortality [Subheading] | 628,303 |
| #5 | Mortality[Mesh] | 422,778 |
| #4 | ((particulate matter [Title/Abstract] OR particulate air pollution [Title/Abstract] OR PM[Title/Abstract] OR PM10[Title/Abstract] OR PM2.5[Title/Abstract]OR particles[Title/Abstract]) OR (Particulate Matter[nm])) OR (Particulate Matter[Mesh]) | 399,232 |
| #3 | particulate matter [Title/Abstract] OR particulate air pollution [Title/Abstract] OR PM[Title/Abstract] OR PM10[Title/Abstract] OR PM2.5[Title/Abstract]OR particles[Title/Abstract] | 349,093 |
| #2 | Particulate Matter[nm] | 28,015 |
| #1 | Particulate Matter [Mesh] | 78,422 |

**Table SA2.** The characteristics of the studies included in the meta-analyses (Umeå, Sweden. 2024).

| **Author, year, study cohort and type** | **Age**  (mean or range) | **Years of follow-up (mean or range)** | **Participants/cases** | **Mean/median PM_2.5_ concentration (µg m^-3^)** | **Effect modification** | **Adjustment** | **Exposure assessment** |
| --- | --- | --- | --- | --- | --- | --- | --- |
| Bauwelinck et al., 2022 [80]  Administrative cohort based on the Belgium 2001 census  Prospective cohort | 52.6 | 9.97 | 5,474,470/  707,138 | 18.73 | Education | Stratified by sex and adjusted for between-area variability by including a cluster term for the neighbourhood. Individual sociodemographic covariates (marital status, country of origin, education level, and occupational status) and area-level socioeconomic position indicators (mean income, unemployment, low education, and ethnicity). | A hybrid land use regression model was employed to estimate pollution exposure at a 100-meter spatial resolution. |
| Brauer et al., 2022 [71]  The Canadian Community Health Survey (CCHS) mortality cohort (mCCHS)  Prospective cohort | 24-89 | 15 | 540,900/  1,551 | 6.97 |  | Model 1: stratified by sex, age (5-year categories), and recent immigrant status, adjusted for income adequacy quintile, visible minority status, indigenous identity, educational attainment, labour-force status, marital status, occupation, and ecological covariates of community size, airshed, urban form, and four dimensions of CAN-Marg (instability, deprivation, dependency, and ethnic concentration).  Model 2: Model 1 + smoking, alcohol consumption, fruit and vegetable con­sumption, BMI, and exercise behaviour. | A hybrid approach was employed to estimate pollution exposure at a 1-km^2^ resolution for individual postal codes by integrating remote sensing, chemical modeling, land use, and ground-level monitoring data. |
| Cesaroni et al, 2013 [65]  Rome Longitudinal Study (RoLS)  Prospective cohort | >30 | 9 | 1,265,058/144,441 |  | Education | Model 1: Sex.  Model 2: Model 1 + marital status, place of birth, education, occupation, and area-based socioeconomic position. | Pollutant exposure at participants’ residential addresses was estimated using dispersion modeling with a 1-km^2^ spatial resolution. |
| Christidis et al., 2019 [66]  The Canadian Community Health Survey (CCHS) mortality cohort (mCCHS)  Prospective cohort |  | 15 | 540,900/  50,700 | 5.9 | Education | Model 1: stratified by age, sex, and cycle, adjusted for visible minority status, indigenous identity, immigrant status, marital status, income quintile, and educational attainment.  Model 2: Model 1 + smoking, alcohol consumption, fruit and vegetable con­sumption, and BMI. | Exposure at postal codes estimated using satellite data, chemical transport models, weighted regression, and ground-level monitoring at 1 km^2^ resolution. |
| Enstrom et al., 2005 [67]  The California Cancer Prevention Study (CA CPS I)  Prospective cohort | 65 | 9 | 49,975/  39,789 | 23.4 | Education | Age, sex,  cigarette smoking status,  race, education level,  marital status,  BMI, male occupational exposure,  exercise, fruit/fruit juice intake, and health status at entry. | County level monitoring data was used to estimate individual exposure. |
| Erickson et al., 2019 [72]  2001 Canadian Census Health and Environment Cohort  Prospective cohort |  | 5.9 | 2,468,190/  196,540 | 8.4 |  | Model 1: stratified by 5-year age-sex groups, adjusted for marital status, visible minority, aboriginal identity, employment, income quintile, education, CAN-Marg index, community size, and airshed.  Model 2: Model 1 + indirectly adjusted for smoking, alcohol use, exercise, and diet. | Exposure was estimated at postal code addresses by integrating satellite-derived PM_2.5_, ground measurements, chemical transport modeling, and weighted regression at 1 km^2^ resolution. |
| Guo et al., 2021 [73]  An open and dynamic cohort without an end date for recruitment or follow-up conducted in Taiwan  Prospective cohort | 39.2 | 13.4 | 842,394/  12,375 | 26.3 |  | Model 1: age, sex, and education.  Model 2: Model 1 + BMI, physical labour at work, cigarette smoking, alcohol drinking, vegetable intake, fruit intake, occupational exposure, season, year of enrolment, and exercise. | Spatiotemporal model was employed to integrate satellite and land base observations to map high resolution air pollution concentration at residential addresses. |
| Hvidtfeldt et al., 2019 [68]  The Danish Diet, Cancer, and Health Cohort  Prospective cohort | 53 | 18.1 | 49,564/  10,193 | 18 | Education | Age, sex, calendar time, educational level, marital status, occupational status, smoking, alcohol consumption, physical activity, BMI, waist circumference, ETS, fruit and vegetable consumption, and neighbourhood-level SES. | Exposure from local, urban, and regional sources estimated at residential addresses using a dispersion model at 1 km^2^ resolution. |
| Nieuwenhuijsen et al., 2018 [74]  SIDAIP cohort based on individuals living in Barcelona  Prospective cohort | 50.9 | 14 | 792,649/  28,391 | 16.08 |  | Model 1: age, gender, and SES.  Model 2: Model 1 + smoking. | Land use regression modeling was employed to assign air pollution concentration at census tract level. |
| Parker et al, 2018 [75]  U.S. National Health Interview Surveys (NHIS)  Prospective cohort | >=25 | 12‒14 | 657,238/  65,936 | 11.8 |  | Model 1: sex, family income as percentage of poverty  threshold, marital status, education, and race/ethnicity. Contextual factors include county-level income, region of the county, and urbanization.  Model 2: Model 1 + BMI and smoking | Monitoring data for individual level exposure. |
| Pope et al., 2019 [69]  U.S. National Health Interview Surveys (NHIS)  Prospective cohort | 43.9 | 17 | 1,599,329/  267,204 | 10.7 | Education, income | Age, sex, race, income, education, marital status, urban versus rural, census regions, survey year, and complex NHIS survey design. | Land use regression estimated air pollution concentrations at participant’s residential addresses. |
| Puett et al., 2009 [76]  Nurses’ Health Study (NHS)  Prospective cohort | 62.4 |  | 66,250/3,785 | 13.9 |  | Model 1: time, residential state, season, family history of MI, hypercholesterolemia, hypertension, BMI, diabetes, median household income, and median house value.  Model 2: Model 1 + smoking. | Exposure estimates were based on urban land use within a 1-km^2^ spatial resolution, buffer zones for point sources (1–10 km), and spatiotemporal GIS modeling. |
| Raaschou-Nielsen et al., 2020 [77]  All Danes born between 1921 and 1985 aged 30-85 years from 1991 to 2015  Nested case-control design | 30-85 | 5.5 | Cases = 672,895  Control = 3,426,533 | 12.7 | Education, income | Model 1: educational level, marital status, occupational status, income, number of children, and country of origin. At neighbourhood level: car ownership, rented dwellings, unemployment, manual professions, education, income, one-parent households, immigrants, and criminal records.  ^*^Model 2: Model 1 + smoking, alcohol consumption, physical activity, BMI, waist circumference, ETS, and fruit and vegetable consumption. | Multiscale dispersion model was used to account for local and regional sources at 1 km^2^ resolution for residential addresses. |
| So et al., 2022 [79]  Danish nationwide  administrative cohort  Prospective cohort | 53 | 15.3 | 3,083,227/803,881 | 12.4 | Education, income | Age (underlying time scale), sex (strata), household income in deciles, occupational status, immigrant status, marital status, highest completed education level, regional mean household income, regional percentage of unemployment, and the difference in mean household income and percentage of unemployment between parish and region. | European-wide hybrid land-use regression estimated the air pollution concentrations at residential addresses with 100-meter spatial resolution. |
| Wang et al., 2023 [78]  Guangzhou cohort  Prospective cohort | 61.2 | 8 | 580,757/37,578 |  | Education | Age, sex, ethnicity, education, marital status, medical insurance, smoking status, exercise frequency, and normalized difference vegetation index (500 m). | Advanced machine learning models integrated satellite-based observations to estimate exposure at residential addresses with 1 km^2^ resolution. |
| Xia et al., 2023 [70]  PURE-China  Prospective cohort | 50.6 | 11.8 | 42,160/2,190 | 45.2 | Education | Model 1: age, sex, baseline year, geographical covariates (urban or rural), region (eastern, central, or western area), and SES (education level and household wealth index).  Model 2: Model 1 + smoking and drinking status, physical activity, fuel for cooking, second-hand smoke exposure, and family history of CVD. | Satellite and ground-based estimates were incorporated by geographically weighted regression model to estimate air pollution concentration at residential addresses. |
| Zhang et al., 2021 [81]  Ontario Health Study (USA)  Prospective cohort | 52.1 |  | 88,615/7,488 | 7.8 |  | Model 1: age, sex, ethnicity, survey year, born in Canada, educational level, marital status, and household income.  Model 2: Model 1 + BMI, fruit and vegetable intake, smoking, alcohol drinking, physical activity, and ETS at home and working place. | Satellite and ground-based observation were incorporated to estimate ground level pollutant concentration to create spatiotemporal surfaces at 1 km^2^ resolution at centroids. |

**Figure SA1.** Funnel plots of PM_2.5_ associated with all-cause mortality (Umeå, Sweden. 2024).

1. by education status


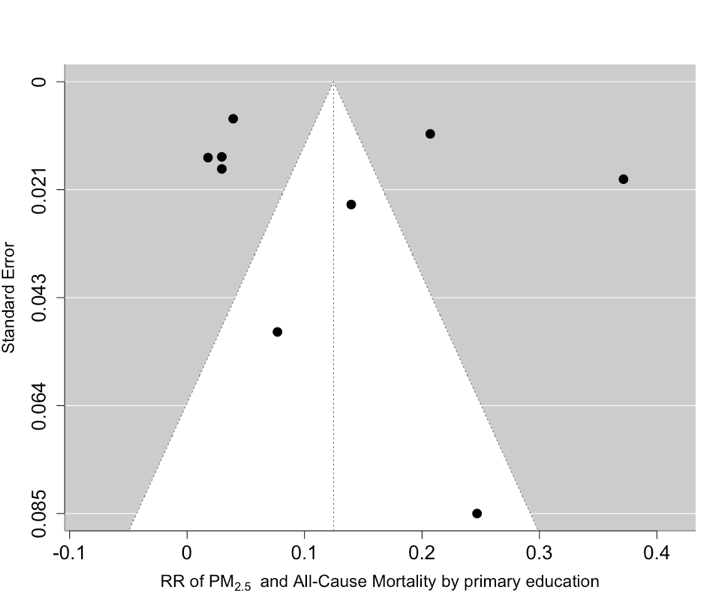

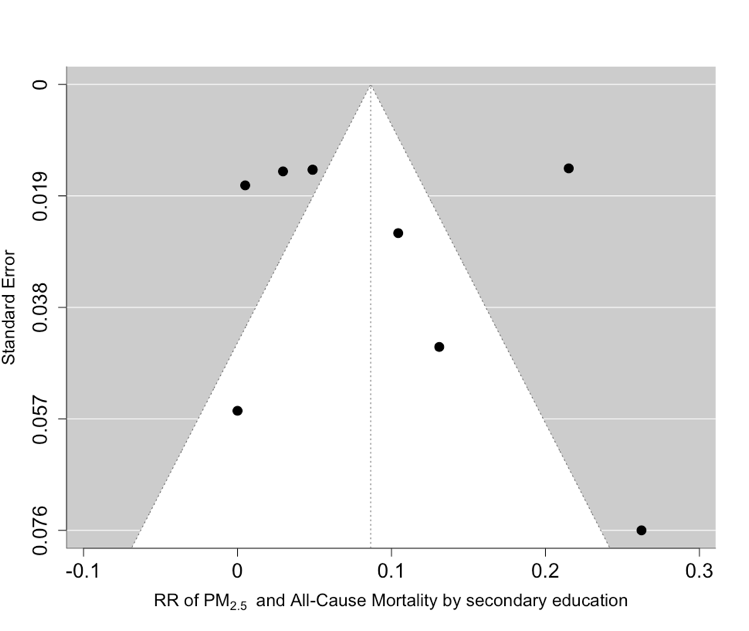

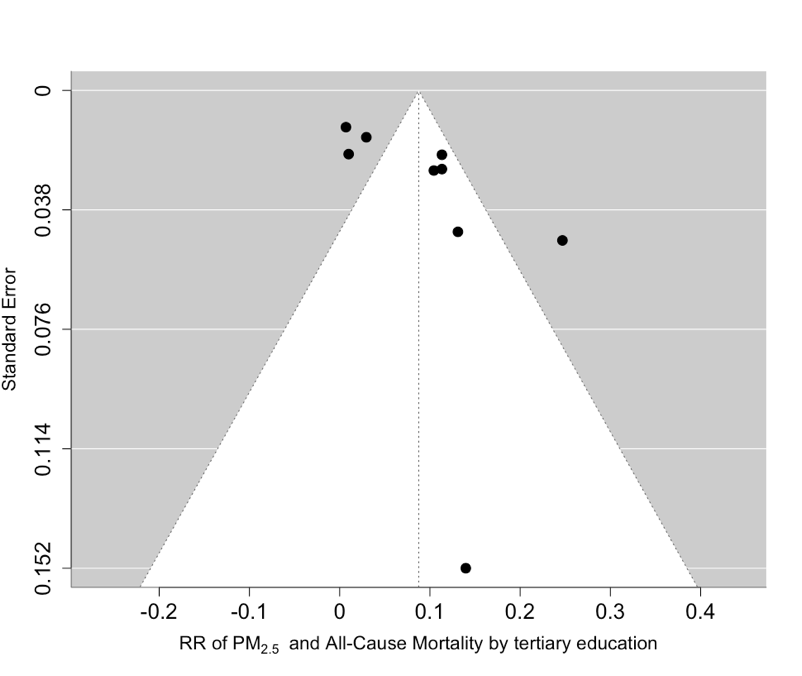


1. by income status


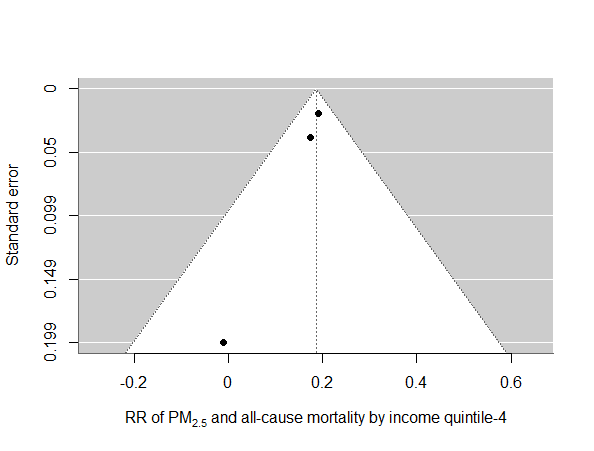

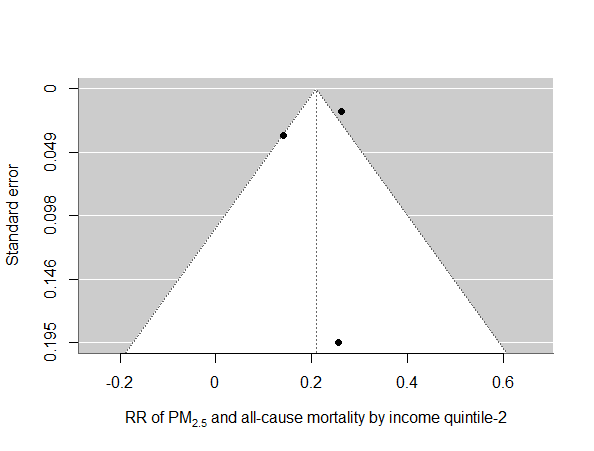

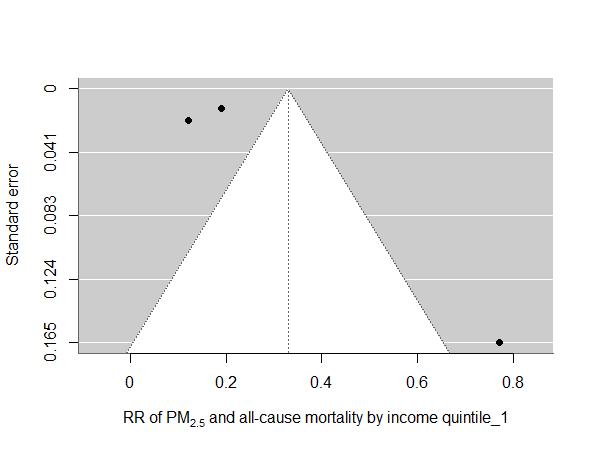

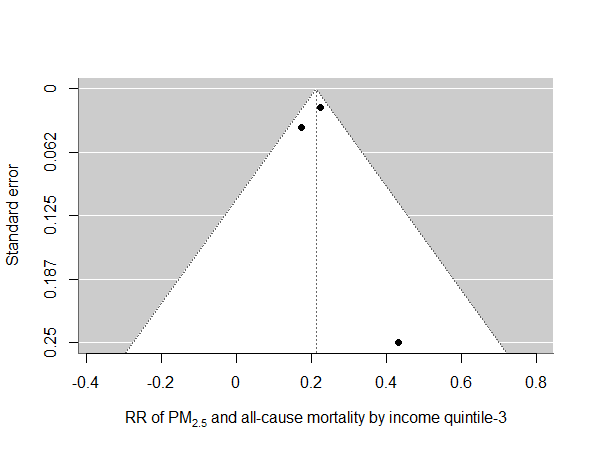

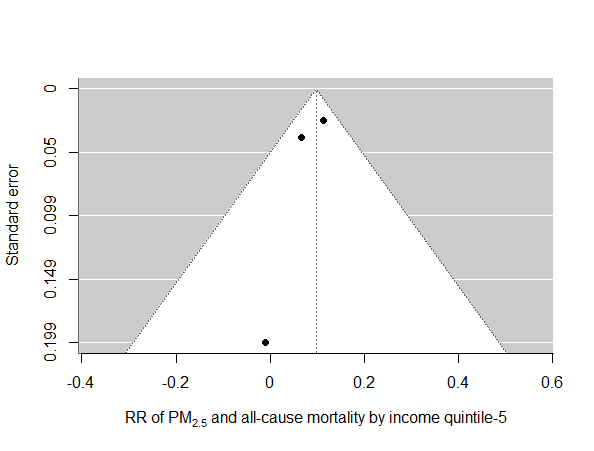


|  |  |
| --- | --- |
|  |  |
|  |  |

c) by adjustment of socioeconomic status and lifestyle factors


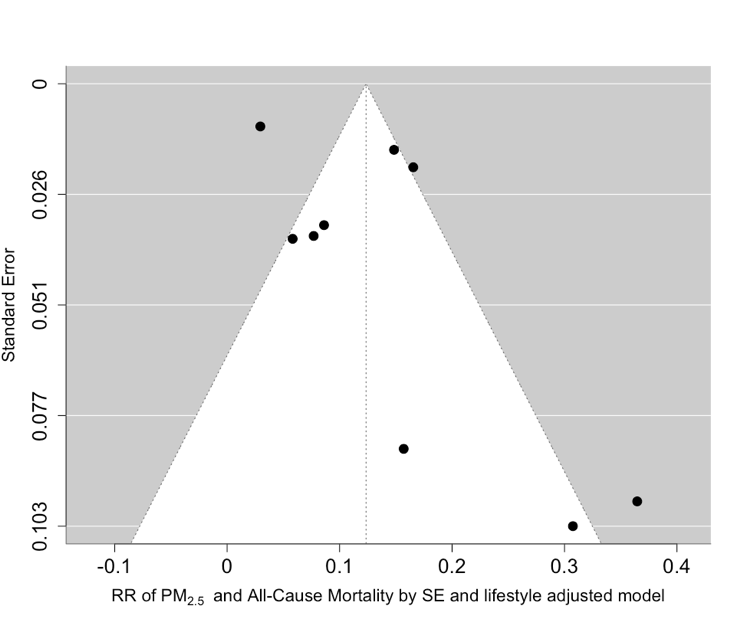

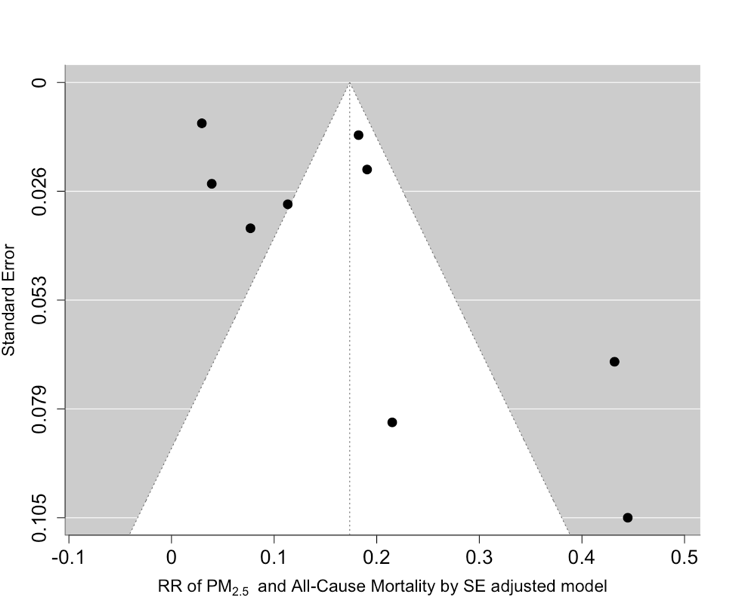

Supplement: Supplementary file 1 [file DataSheet1.docx]
